# Crystallization Behavior and Mechanical Property of Biodegradable Poly(butylene succinate-*co*-2-methyl succinate)/Cellulose Nanocrystals Composites

**DOI:** 10.3390/polym16121735

**Published:** 2024-06-19

**Authors:** Wenxin Yao, Siyu Pan, Zhaobin Qiu

**Affiliations:** State Key Laboratory of Chemical Resource Engineering, Beijing University of Chemical Technology, Beijing 100029, China; 2022200363@grad.buct.edu.cn (W.Y.); 2022400087@grad.buct.edu.cn (S.P.)

**Keywords:** biodegradable, cellulose nanocrystals, composites, crystallization

## Abstract

Biodegradable poly(butylene succinate-*co*-2-methyl succinate) (PBSMS)/cellulose nanocrystals (CNC) composites were successfully prepared at low CNC loadings with the aims of improving crystallization and mechanical properties and extending the practical application of PBSMS. CNC is finely dispersed in the PBSMS matrix without obvious aggregations. The low content of CNC obviously promoted the crystallization behavior of PBSMS under different conditions. The spherulitic morphology study revealed that CNC, as an effective heterogeneous nucleating agent, provided more nucleation sites during the melt crystallization process. In addition, the nucleation effect of CNC was quantitatively evaluated by the following two parameters, i.e., nucleation activity and nucleation efficiency. The crystal structure and crystallization mechanism of PBSMS remained unchanged in the composites. In addition, as a reinforcing nanofiller, CNC significantly increased Young’s modulus and the yield strength of PBSMS. The crystallization behavior and mechanical properties of PBSMS were significantly improved by the low content of CNC, which should be interesting and essential from the perspective of biodegradable polymer composites.

## 1. Introduction

The excessive use of traditional petroleum-based nondegradable polyesters has recently caused serious issues to the ecological environment [1]. Therefore, many researchers have developed various biodegradable polyesters to replace traditional petroleum-based polyesters, such as polylactic acid (PLA), polyhydroxyalkonoates (PHAs), and poly(butylene adipate-*co*-terephthalate) (PBAT) [2,3,4,5,6]. Among these polyesters, poly(butylene succinate) (PBS) has received extensive attention from both academia and industry due to its excellent thermal and mechanical properties and easy processability [4,5,6]. However, the relatively low degradability rate and poor ductility of PBS hinder its practical and wide-ranging applications [5,6]. To improve the comprehensive physical properties, PBS has often been modified by a copolymerization method. For instance, Zheng et al. used PBS and polycaprolactone (PCL) diol segments to prepare some copolymers with desirable mechanical properties and double crystalline multiblock via a chain extension reaction [7]. In addition, the introduction of the 1,6-hexanediol, 2,3-butylene, and 1,3/1,4-CHDM comonomers into PBS may also enhance the elongation at the break and toughness of PBS-based copolymers [8,9,10]. In the literature, Guo et al. found that the introduction of the unsaturated fumaric acid containing trans double-bond significantly increased the crystallization rate of PBS; moreover, poly(butylene succinate-*co*-butylene fumarate) (PBSF) copolymers exhibited an interesting isomorphism phenomenon [11].

In addition, 2-methyl succinic acid (2-MSA), a biobased monomer from itaconic acid, was also used to modify PBS to maintain the same backbone as PBS [12,13,14]. In 2004, Chea et al. synthesized novel PBS-based copolymers with butylene 2-methyl succinate (BMS) unit from 5 to 20 mol%, i.e., poly(butylene succinate-*co*-2-methyl succinate) (PBSMS). They found that the methyl substitution obviously retarded the crystallization of PBSMS; however, the toughness was notability improved [12]. In 2019, Han et al. further synthesized PBSMS copolymers with BMS unit content from 10 to 50 mol%. With increasing the molar ratio of the BMS unit, the crystallization behavior of PBSMS showed the same trend as the above research results reported by Chea et al.; furthermore, PBSMS displayed better biodegradability than PBS under the same enzymatic degradation condition [13]. Recently, we synthesized the PBSMS random copolymers with BMS unit from 10 to 90 mol% and systematically investigated the effects of different contents of BMS unit on the crystallization behavior, spherulitic morphology and growth rate, crystallization regime transition, and mechanical property of PBSMS. Compared with that of PBS, the elongation at break of PBSMS was significantly improved; however, both the crystallization ability and the tensile strength of PBSMS gradually weakened with increasing BMS unit content. When the molar ratio of the BMS unit was higher than 50 mol%, PBSMS even became amorphous and could not crystallize [14]. Among these PBSMS copolyesters, PBSMS20 with a 20 mol% of BMS unit was of great interest and importance due to its balanced thermal and mechanical properties with a melting point of about 96 °C and an elongation at break of 333.3 ± 54.6% [14]; however, its crystallization rate and tensile mechanical property (including Young’s modulus, yield strength, and tensile strength) should be further improved for its wider practical application.

Cellulose crystals (CNC) are a kind of biobased cellulose-derived nanofiller with high tensile modulus, crystallinity, and specific surface area [15]. Thus, CNC has been widely used in the preparation of polymer/CNC composites with the purpose of accelerating the crystallization process and improving the mechanical properties of polymers [16,17,18,19]. In the literature, CNC has already been used to modify biodegradable and/or biobased polymers, such as PLA, PCL, PHAs, PBS, and some furan-based homopolymers [20,21,22,23,24,25,26,27,28,29,30,31,32,33,34,35,36,37,38,39]. The above studies clearly show that the crystallization and mechanical properties of these polymers could be simultaneously improved by CNC, thus facilitating the practical application of these biodegradable and/or biobased polyesters [20,21,22,23,24,25,26,27,28,29,30,31,32,33,34,35,36,37,38,39].

To solve the issues of the slow crystallization rate and low mechanical strength of PBSMS copolyester, a small content of CNC was introduced via a solution and casting method in this research. The finely dispersed CNC significantly promoted the melt crystallization behavior under both the nonisothermal and isothermal melt crystallization conditions, while the crystal structure of PBSMS/CNC composites remained unchanged. Moreover, the tensile test results showed that CNC remarkably improved the Young’s modulus and tensile strength of PBSMS. The research results are expected to be interesting and important in both polymer crystallization and biodegradable polymer composites fields.

## 2. Experimental

### 2.1. Materials and Preparation of PBSMS/CNC Composites

PBSMS ([*ƞ*] = 0.84 dL/g, BMS unit content = 20 mol%) was synthesized by our laboratory through a two-step melt polycondensation method [14]. It should be noted that the weight-average molecular weight and number-average molecular weight of PBSMS could be measured with gel permeation chromatography if chloroform was used as the solvent. CNC (about 5–20 nm in diameter and 50–200 nm in length) was obtained from Shanghai ScienceK Nanotechnology Co., Ltd. (Shanghai, China). N, N-dimethylformamide (DMF) (purity = 99.5%) was supplied by Tianjin Damao Chemical Reagent Factory (Tianjin, China).

For brevity, the preparation process of PBSMS/CNC composites is shown in the Supplementary Materials. In this research, PBSMS/CNC composites were prepared using N, N-dimethylformamide (DMF) as a solvent with the help of sonication. Although DMF was a high boiling point solvent, it could be completely removed. As we evaporated the mixture in a fume hood to form a film, most DMF was evaporated during this process. The residual was further evaporated in a vacuum oven at 50 °C for 7 days. When measured with thermogravimetric analysis, we could not see any trace of DMF, confirming the complete removal of DMF.

**Scheme 1:**
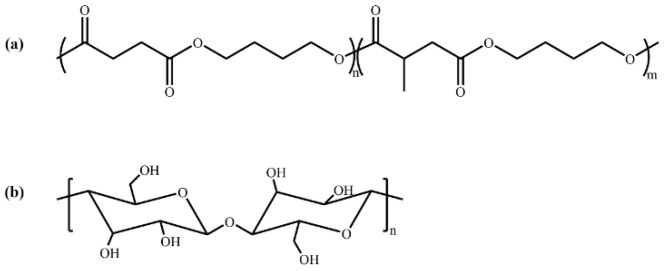
Chemical structures of (**a**) PBSMS and (**b**) CNC.

Scheme 1 illustrates the chemical structures of PBSMS and CNC as follows.

### 2.2. Characterizations

A scanning electron microscope (SEM) (JEOL, JSM-7800, Tokyo, Japan) was used to study the distribution of CNC in PBSMS. All samples were fractured in liquid nitrogen. The fractured surfaces were coated with gold before observation.

**Figure 1:**
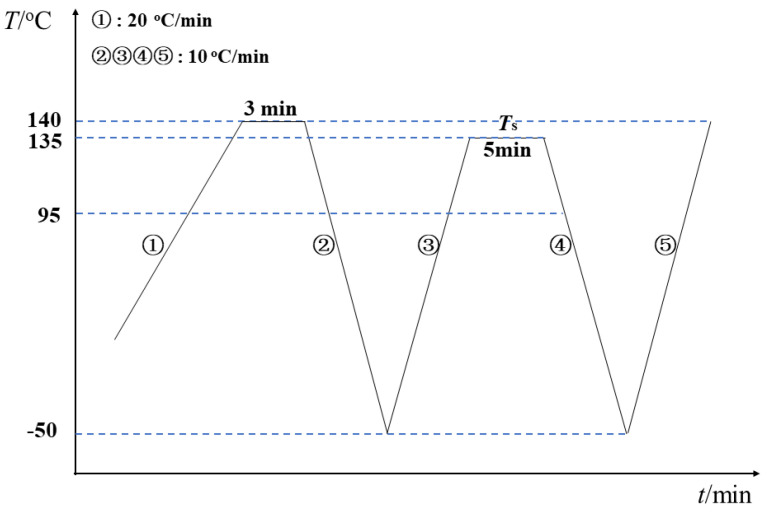
The standard self-nucleation procedure.

The crystallization behavior of all samples was investigated using a differential scanning calorimeter (DSC) (TA Instrument Q100, New Castle, DE, USA) under the nitrogen atmosphere. The mass of the sample was approximately 4–5 mg and sealed in an aluminum crucible. For the study of nonisothermal crystallization behavior, DSC was performed to cool at different rates (5~25 °C/min) after eliminating the thermal history at 140 °C for PBSMS and its composites. For the study of isothermal crystallization kinetics, the sample was cooled at 60 °C/min to the isothermal crystallization temperature after erasing the thermal history. The standard DSC thermal procedure was performed to study the self-nucleation of PBSMS, as shown in Figure 1. Finally, the untested mechanical samples were heated in DSC at 10 °C/min to measure the melting enthalpies.

The spherulitic morphology of all samples was observed using polarizing optical microscopy (POM) (Olympus BX51, Tokyo, Japan) with the temperature controller (Linkam THMS 600, Redhil, UK). The samples were quenched to 72 °C after eliminating the thermal history and continued to be observed until the spherulites grew and filled the field of view. Additionally, PBSMS were cooled at 10 °C/min from 135 °C (self-nucleation, domain I) and 98 °C (domain II) to 35 °C, respectively.

The crystal structures of the samples were investigated by a Rigaku Ultima IV X-ray diffractometer from 5° to 50° at 5°/min, and the CuKα radiation (λ = 0.15418 nm) source was operated at 40 kV and 200 mA. The samples were annealed at 50 °C for 24 h before the wide-angle X-ray diffraction (WAXD) test. In addition, from the WAXD pattern, the degree of crystallinity was also obtained by the ratio of the area belonging to the crystalline region to the sum of that of the crystalline region and that of the amorphous region using the well-known Jade software.

The mechanical properties were studied with a UTM5205XHD instrument (SUNS, Zhangzhou, China) at a crosshead speed of 50 mm/min. Five dumbbell-shaped specimens were measured for each sample.

The standard self-nucleation procedure in Figure 1 is described as follows:

1. The sample was melted at 140 °C for 3 min to remove the previous thermal history.
2. The creation of the standard semicrystalline state was achieved by cooling the meltdown to −50 °C at a rate of 10 °C/min.
3. Then, the sample was heated to an indicated self-nucleation temperature (*T*_s_) at a rate of 10 °C/min and held at this temperature for 5 min. The self-nucleation temperature determined the various states that the sample went through, such as melting, self-nucleation, and annealing.
4. The subsequent crystallization further occurred by cooling the sample at a rate of 10 °C/min.
5. The sample was finally heated again to 140 °C at 10 °C/min to investigate the melting behavior.

## 3. Result and Discussion

### 3.1. Dispersion of CNC in PBSMS Matrix

**Figure 2:**
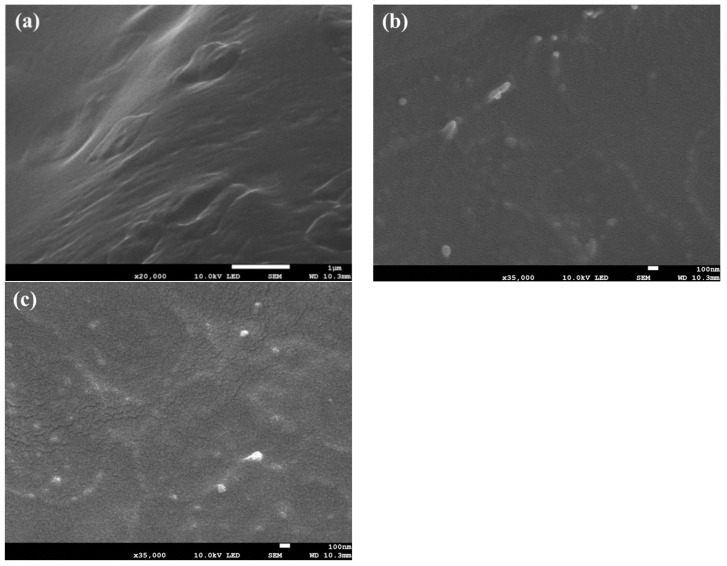
Fractured surface morphology images of (**a**) PBSMS, (**b**) PBSMS/CNC0.5, and (**c**) PBSMS/CNC1.

It is well-known that the morphology and dispersion of nanofillers in the polymer matrix are essential to the improvement of the physical properties of the composites [17]. SEM was, therefore, first used in this research to study the morphology and dispersion of CNC in the PBSMS matrix. From Figure 2, CNC was randomly dispersed in the PBSMS matrix, suggesting an even dispersion of CNC in the two composites.

### 3.2. Nonisothermal Melt Crystallization Behavior Study

**Figure 3:**
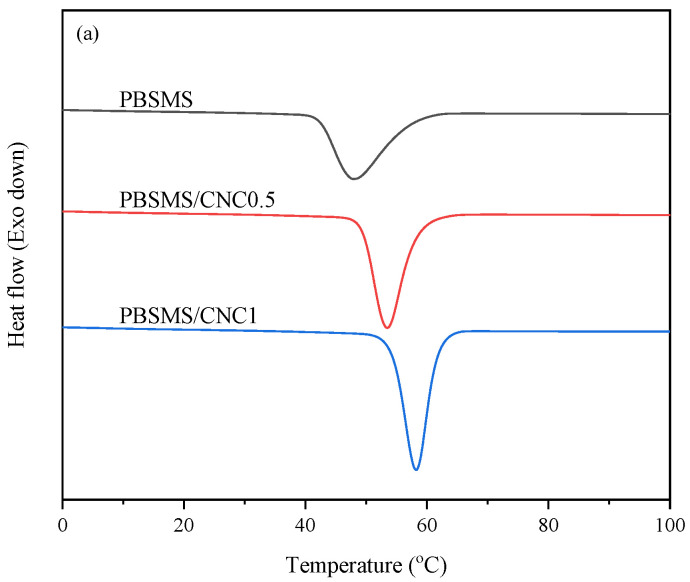

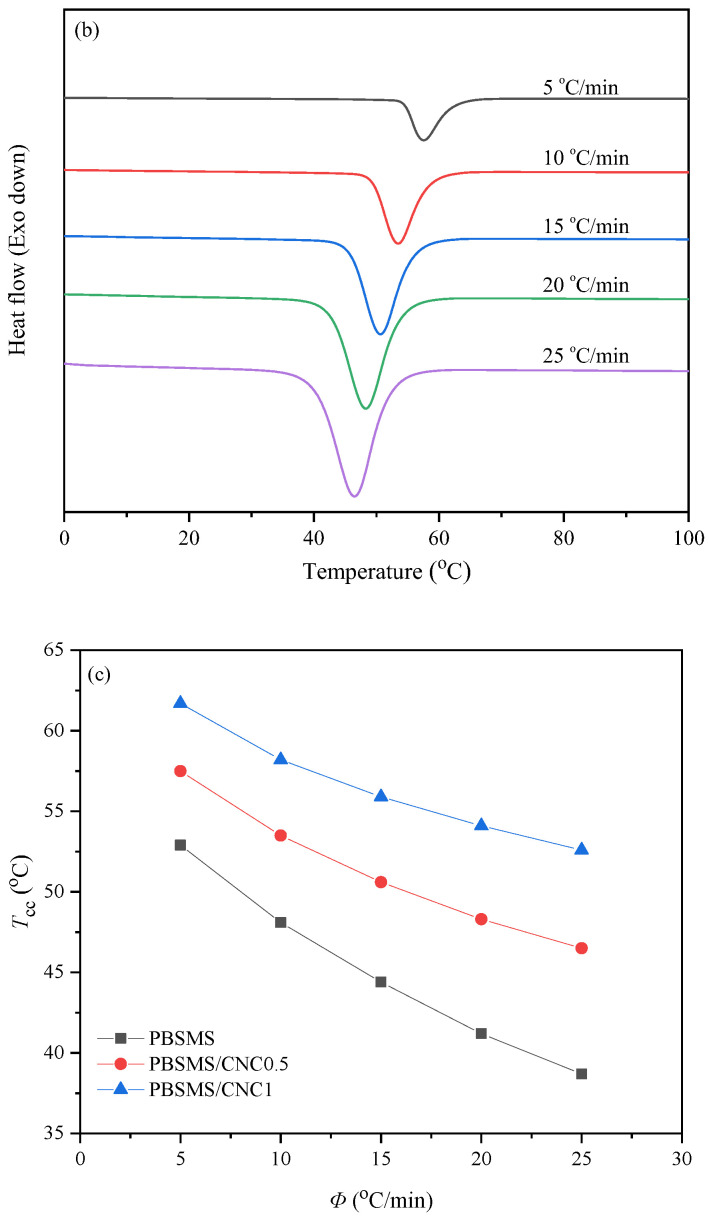
(**a**) Nonisothermal melt crystallization behavior of PBSMS and its composites at 10 °C/min, (**b**) nonisothermal melt crystallization behavior of PBSMS/CNC0.5 at different cooling rates, and (**c**) variation of *T*_cc_ with *Φ* for PBSMS and its composites.

The nonisothermal melt crystallization behavior was first studied for PBSMS and PBSMS/CNC composites. Figure 3a displays the DSC cooling traces of PBSMS and its composites at 10 °C/min. PBSMS exhibited a relatively broad crystallization exothermic peak at 48.1 °C with an enthalpy of 58.7 J/g. In the case of the two composites, it became sharper and shifted upwards to higher temperatures. The melt crystallization temperature (*T*_cc_) and melt crystallization enthalpy of the composites significantly increased to 53.5 °C and 64.7 J/g for PBSMS/CNC0.5 and 58.2 °C and 65.6 J/g for PBSMS/CNC1, respectively. As a result, CNC greatly enhanced the nonisothermal melt crystallization of PBSMS as an efficient biobased and biodegradable nucleating agent.

The influence of different cooling rates (*Φ*) on the nonisothermal melt crystallization behavior of all samples was further studied. Figure 3b exhibits the DSC cooling traces of PBSMS/CNC0.5 at different rates. With decreasing *Φ* from 25 to 5 °C/min, *T*_cc_ of PBSMS/CNC0.5 gradually increased from 38.7 to 52.9 °C. The obvious increase in *T*_cc_ should be attributed to the fact that the time was longer enough for the samples to nucleate and grow at a lower cooling rate. For brevity, Figure S1 in the Supplementary Materials depicts the DSC cooling traces of PBSMS and PBSMS/CNC1. A similar trend was observed for PBSMS and PBSMS/CNC1, as shown in Figure S1. For a better comparison, the variation of *T*_cc_ with *Φ* is shown in Figure 3c. As *Φ* decreased, *T*_cc_ gradually increased for all samples. At the same *Φ*, the *T*_cc_ of PBSMS obviously shifted upwards to a high-temperature range with the increasing CNC content.

### 3.3. Isothermal Crystallization Kinetics Study

**Figure 4:**
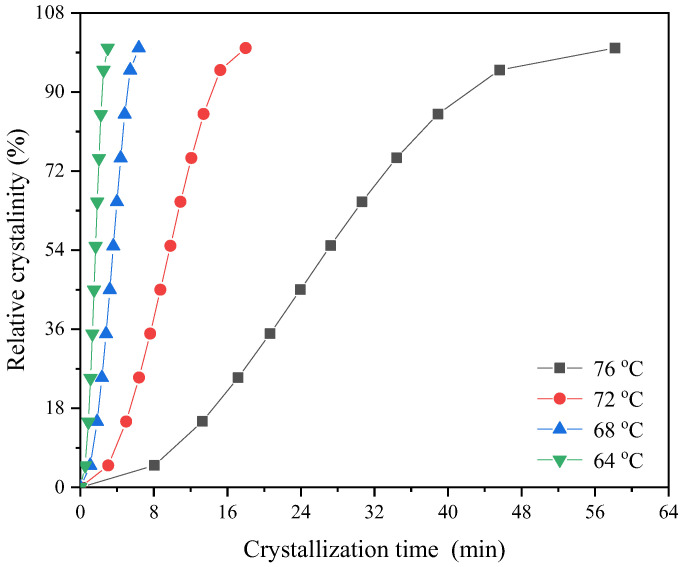
Plots of relative crystallinity versus crystallization time for PBSMS/CNC0.5.

The isothermal melt crystallization kinetics of PBSMS and its composites were also studied with DSC. For instance, the original heat flow evolution with time during the isothermal crystallization at 76 °C is illustrated in Figure S2 for PBSMS and its composites in the Supplementary Materials, from which the crystallization time of PBSMS significantly became shorter by the presence of CNC as a nucleating agent. CNC reduced not only the crystallization induction time but also the total crystallization time of PBSMS. Figure 4 displays the development of relative crystallinity (*X*_t_) with crystallization time (*t*) for PBSMS/CNC0.5 at different crystallization temperature (*T*_c_) values. As shown in Figure 4, crystallization time significantly became longer with increasing *T*_c_, indicating the slower crystallization rate due to the smaller degree of supercooling. For instance, with increasing *T*_c_ from 64 to 76 °C, the crystallization time of PBSMS/CNC0.5 greatly increased from 3.0 to 50.4 min. A similar trend was observed for PBSMS and PBSMS/CNC1, as shown in Figure S3 in the Supplementary Materials.

The isothermal crystallization kinetics of PBSMS and PBSMS/CNC composites were further analyzed by the classical Avrami equation [40,41,42]. The relationship between *X*_t_ and *t* was described as follows:

(1) $$1-X_{t}=\exp\;(-kt^{n})$$

where *n* represents the Avrami exponent, and *k* is the crystallization rate constant.

**Figure 5:**
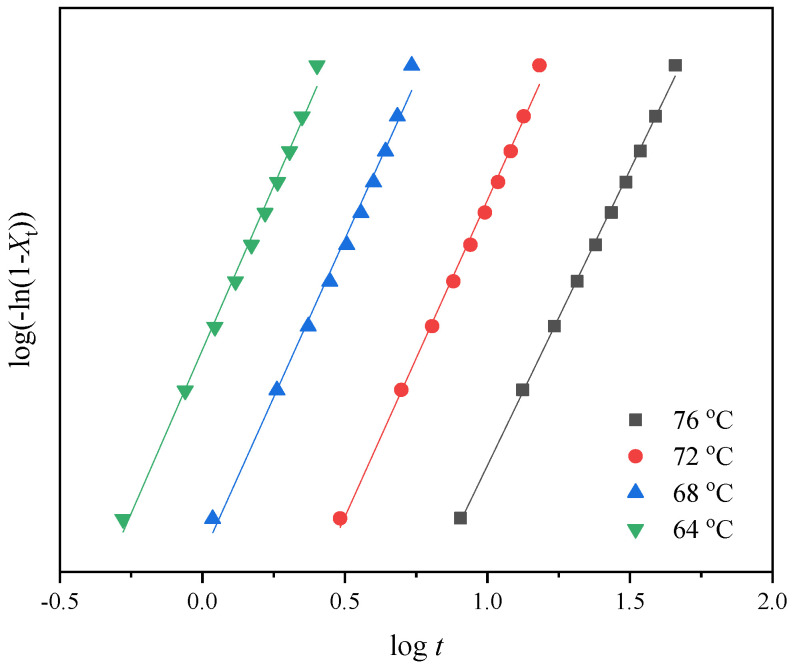
Avrami plots of PBSMS/CNC0.5.

Figure 5 presents the Avrami plots of PBSMS/CNC0.5 at different *T*_c_ values. There were four almost parallel fitting lines, indicating the applicability of the Avrami equation. Figure S4 depicts the Avrami plots of PBSMS and PBSMS/CNC1, showing similar results. Table 1 lists the Avrami parameters *n* and *k* for all samples. For both PBSMS and its composites, the *n* values slightly varied between 2.2 and 2.7, suggesting that PBSMS and its composites should crystallize through the same three-dimensional spherical growth with an athermal nucleation mechanism [43].

The *k* values apparently increased with a decrease in *T*_c_ or an increase in CNC loading, suggesting a faster crystallization rate. In addition, the crystallization half-time (*t*_0.5_) was used to directly compare the crystallization rate, which corresponded to the time to complete 50% of the final crystallinity and was calculated as follows:

(2) t0.5=(ln2k)1n

**Figure 6:**
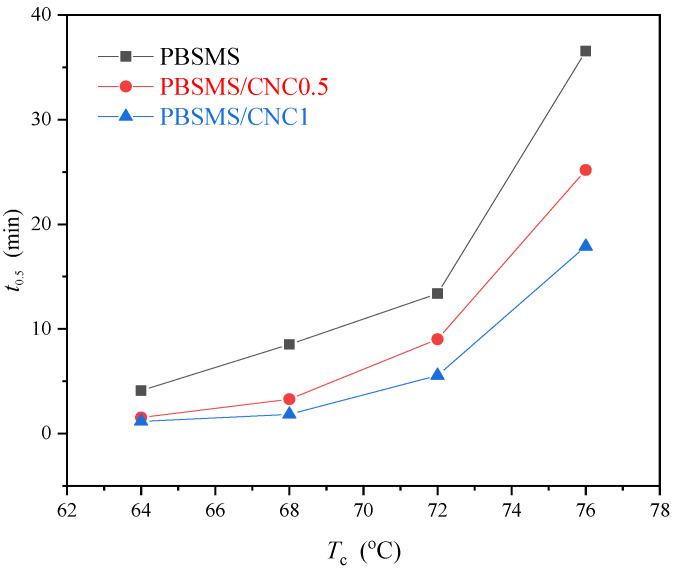
Variation of *t*_0.5_ with *T*_c_ for PBSMS and its composites.

The obtained *t*_0.5_ values are also listed in Table 1. Figure 6 illustrates the variations of *t*_0.5_ with *T*_c_ for PBSMS and its composites. For each sample, the *t*_0.5_ values obviously increased with increasing *T*_c_, indicating a slower crystallization rate. Moreover, at the same *T*_c_*, t*_0.5_ remarkably decreased with an increase in CNC loading. For instance, at a *T*_c_ of 76 °C, the *t*_0.5_ values decreased greatly from 36.6 min for PBSMS to 25.2 min for PBSMS/CNC0.5 and 17.9 min for PBSMS/CNC1, respectively, revealing that the isothermal melt crystallization of PBSMS was obviously accelerated by CNC. It could, thus, be concluded from the above studies that CNC acted as an efficient heterogeneous nucleating agent and promoted the melt crystallization of PBSMS at the same cooling rate or at the same crystallization temperature.

### 3.4. Nucleation Study

**Figure 7:**
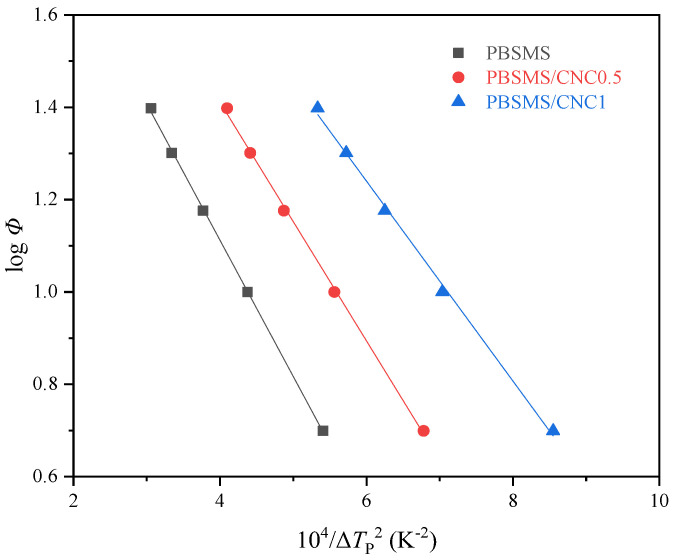
Plots of log *Φ* versus 10^4^/Δ*T*_p_^2^ for PBSMS and its composites.

The nucleation effect of CNC on the crystallization of PBSMS was further quantitatively evaluated in this section. At first, the nucleation activity (*N*_a_) was determined by the following equations [44,45]:

(3) $$\log\;\Phi =A\;-\;B\text{∕}\;(2.3\Delta T_{p}^{2})$$

(4) $$\log\;\Phi =A\;-\;B^{*}\text{∕}\;(2.3\Delta T_{p}^{2})$$

where ∆*T*_p_ is the degree of supercooling, and *A*, *B*, and B* are constants. Equations (3) and (4) are suitable for homogeneous nucleation and heterogeneous nucleation, respectively; furthermore, *N*_a_ was derived from the ratio of B*/*B*. The variation of log*Φ* with 10^4^/Δ*T*_p_^2^ is exhibited in Figure 7, from which the *N*_a_ values were derived to be 0.83 and 0.70 for PBSMS/CNC0.5 and PBSMS/CNC1, respectively. In a previous study, the *N*_a_ value of poly(butylene succinate-*co*-1,2-decylene succinate) (PBDS)/CNC1 (the content of CNC = 1 wt%) was 0.52, **demonstrating that CNC displayed a stronger nucleation activity in PBDS than in PBSMS [37].**

The nucleation effect of CNC in the PBSMS matrix was further quantitatively analyzed by the nucleation efficiency (*NE*). The calculation equation is as follows:

(5) $$NE=\frac{T_{\mathrm{cn}}-T_{\mathrm{cc}}}{T_{\mathrm{cs}}-T_{\mathrm{cc}}}$$

where *T*_cc_ and *T*_cn_ are the melt crystallization temperatures of PBSMS and PBSMS/CNC composites at 10 °C/min, respectively, while *T*_cs_ is the melt crystallization temperature of PBSMS at the ideal self-nucleation temperature obtained through a self-nucleation study [46,47].

**Figure 8:**
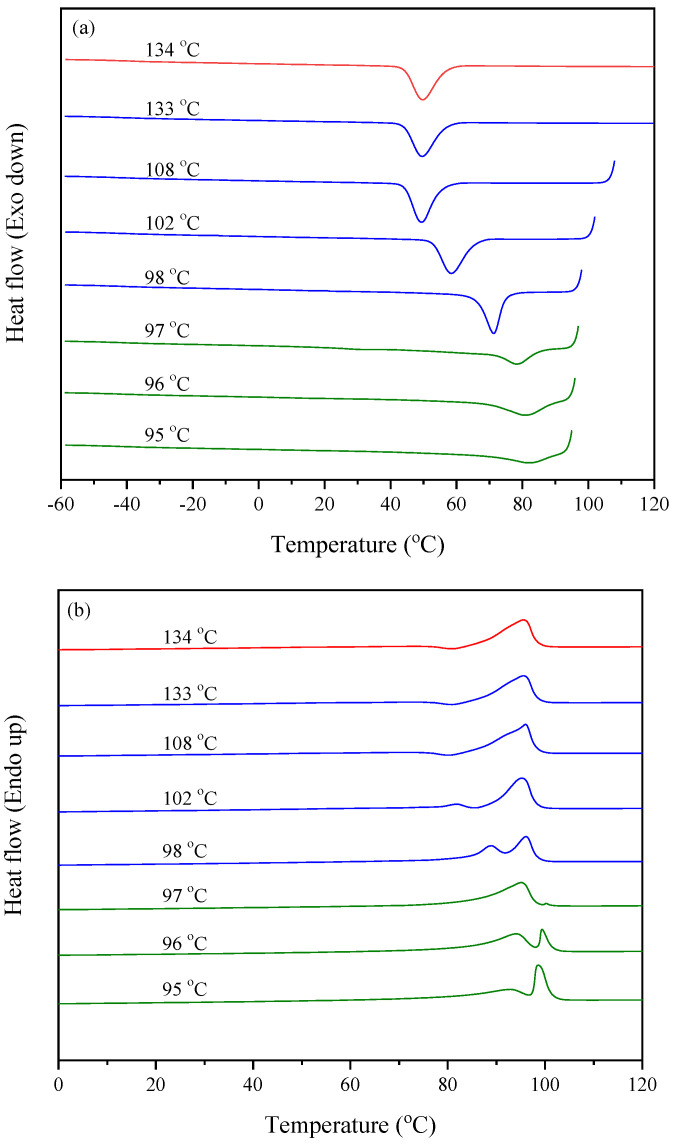
Self-nucleation of PBSMS: (**a**) melt crystallization from the indicated *T*_s_ and (**b**) subsequent melting behavior (cooling and heating at 10 °C/min).

To determine the *T*_cs_ value of PBSMS, we performed a standard self-nucleation procedure study in this section. The detailed self-nucleation procedure is shown in Figure 1. As proposed by Fillon et al., the self-nucleation process could be divided into three domains [46,47]. From Figure 8, the thermal history of PBSMS was completely erased in domain I (*T*_s_ ≥ 134 °C); consequently, *T*_cc_ remained almost unchanged at about 49 °C. In domain II (133 °C ≤ *T*_s_ ≤ 98 °C), *T*_cc_ shifted to a higher temperature (49.7 °C–71.3 °C), suggesting that PBSMS underwent a self-nucleation process. Certain melt memory or some crystal fragments of PBSMS acted as self-nuclei or self-seeds, facilitating the subsequent cooling process within this *T*_s_ range, resulting in an increase in *T*_cc_ [48]. In domain III (*T*_s_ ≤ 97 °C), *T*_cc_ further increased; furthermore, a new higher melting peak appeared beside the main melting peak at around 99 °C, suggesting the simultaneous occurrence of both self-nucleation and annealing.

The ideal self-nucleation temperature of PBSMS, the lowest temperature in domain II, was, thus, determined to be 98 °C. Therefore, the *T*_cs_ value of PBSMS was determined to be 71.3 °C. The *NE* values were calculated to be 23.3% and 43.5% for PBSMS/CNC0.5 and PBSMS/CNC1, respectively. In the literature, the *NE* values were 10% in PCL, 42.9% in poly(hexamethylene 2,5-furandicarboxylate) (PHF), and 44.9% in PBDS, respectively, when the same CNC content of 1 wt% was used [24,37,38]. In brief, CNC exhibited relatively strong nucleation effects in PBSMS, which were comparable to those in other biodegradable or biobased polyesters [24,37,38].

**Figure 9:**
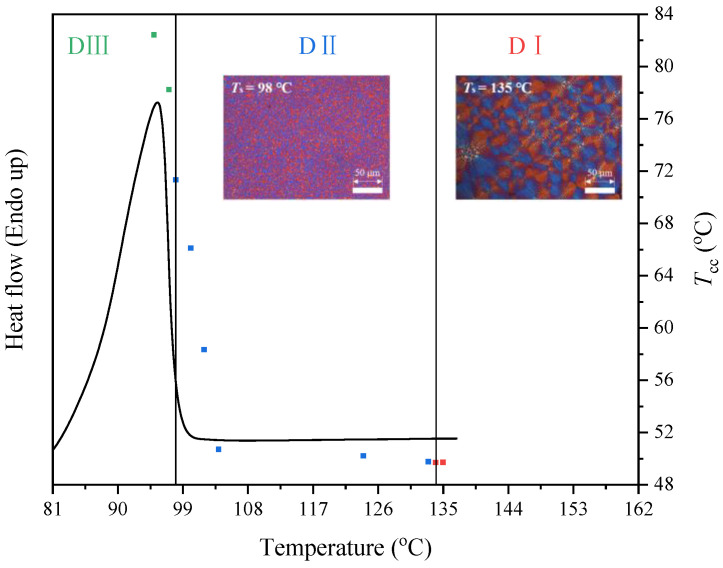
The melting behavior of PBSMS and the point data represent the *T*_cs_ at indicated *T*_s_ values. (The inserted POM images of PBSMS spherulites were cooled from domain I or II).

Figure 9 displays the plots of *T*_cc_ versus *T*_s_, while the illustration curve is the DSC heating scan of PBSMS at 10 °C/min. The variation of *T*_cc_ with *T*_s_ was consistent with the previous description. In addition, the spherulitic morphology of PBSMS is also shown in Figure 9 at 35 °C after cooling at the rate of 10 °C/min from 135 °C (domain I) or 98 °C (domain II), respectively. The spherulite density cooled from domain II was extremely higher than that cooled from domain I, directly revealing the effect of the presence of the melt memory or the crystal fragments in domain II on the subsequent crystallization of PBSMS.

### 3.5. Crystal Structure and Spherulitic Morphology Studies

**Figure 10:**
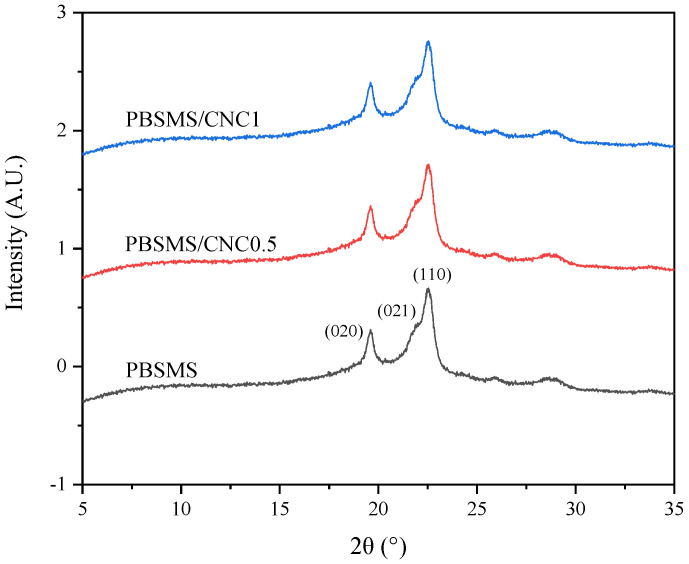
WAXD profiles of PBSMS and its composites.

The crystal structure of PBSMS and PBSMS/CNC composites was further studied. The WAXD patterns are presented in Figure 10. All samples showed similar three main diffraction peaks at 2*θ* = 19.4°, 21.7°, and 22.4°, which were attributed to (020), (021), and (110) planes of PBS, respectively, revealing that CNC did not modify the crystal structure of PBSMS [49,50]. Additionally, the crystallinity values were estimated from the WAXD patterns, which were around 42%, 45%, and 47% for PBSMS, PBSMS/CNC0.5, and PBSMS/CNC1, respectively. Similar to the DSC study above, the crystallinity of PBSMS also increased with an increase in CNC content. However, due to the different crystallization conditions, the effect of CNC on the increase in crystallinity of PBSMS was not the same. In the case of the DSC study, the crystallization occurred fast within a short period; therefore, the effect of CNC was obvious. In the case of the WAXD study, the crystallization time was very long; therefore, the effect of CNC was slight. In brief, the presence of CNC did not modify the crystal structure of PBSMS in the composites, while the crystallinity slightly increased.

**Figure 11:**
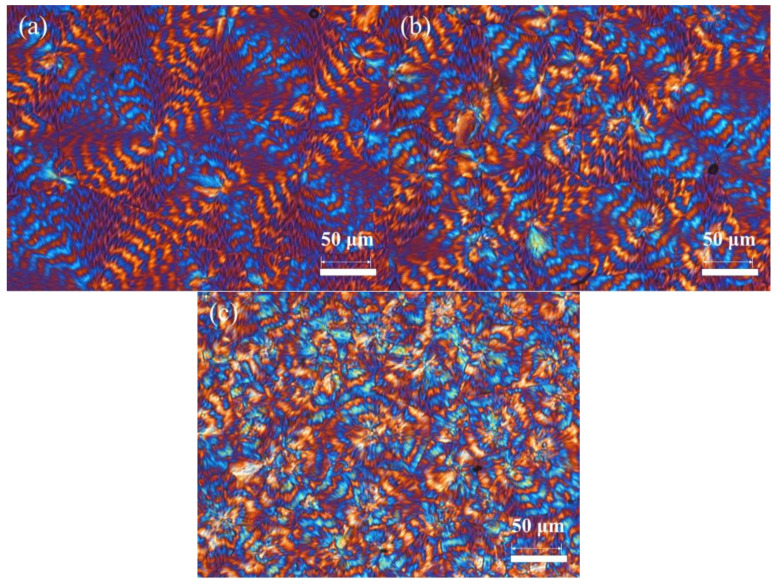
Spherulitic morphology of (**a**) PBSMS, (**b**) PBSMS/CNC0.5, and (**c**) PBSMS/CNC1. (the same scalar bar).

The influence of CNC on the spherulitic morphology of PBSMS was investigated with POM. Figure 11 illustrates the POM images of PBSMS and PBSMS/CNC composites after completely isothermally crystallizing at 72 °C. Banded spherulites were observed for both PBSMS and its composites at this temperature. In addition, the nucleation density of PBSMS spherulites remarkably increased in the composites, especially in PBSMS/CNC1. The spherulitic morphology study directly showed the nucleating agent role of CNC, which provided additional sites for PBSMS to nucleate and grow in the composites.

### 3.6. Mechanical Property Study

**Figure 12:**
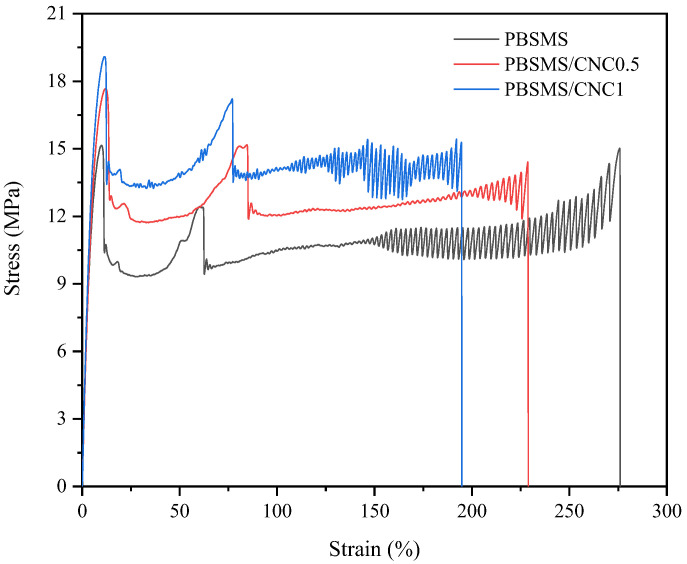
Stress–strain curves of PBSMS and its composites.

The mechanical property of PBSMS and its composites was further measured via tensile tests. The typical stress–strain curves of the three samples are depicted in Figure 12, demonstrating obvious plastic deformation and yielding behavior. Table 2 lists the detailed tensile test data. On the one hand, with increasing CNC loading, Young’s modulus (*E*_t_) and yield strength (*σ*_y_) significantly increased from 242.9 ± 12.4 and 10.6 ± 1.3 MPa for PBSMS to 320.3 ± 14.6 and 16.5 ± 0.9 MPa for PBSMS/CNC0.5 and 321.3 ± 5.14 and 19.1 ± 0.5 MPa for PBSMS/CNC1, respectively. The mechanical property of the composites was apparently improved due to the inherent rigidity of CNC and the increase in the crystallinity of composites. From Figure S5, the melting enthalpies (Δ*H*_m_) significantly increased from 46 J/g for PBSMS to 60 and 64 J/g for PBSMS/CNC0.5 and PBSMS/CNC1, respectively, indicating that the crystallinity of the composites remarkably increased by the presence of CNC. On the other hand, the elongation at break (*ε*_b_) accordingly declined from 333.3 ± 54.6% for PBSMS to 231.9 ± 15.6% and 198.6 ± 14.7% for PBSMC/CNC0.5 and PBSMS/CNC1, respectively; however, the *ε*_b_ values were still relatively high in the composites, about 200% or above. The presence of CNC may lead to the concentration of stress in the PBSMS matrix, in which cracks were more likely to start and develop. In brief, the obvious increase in *E*_t_ and *σ*_y_ clearly indicated that the low content of CNC dramatically reinforced PBSMS.

## 4. Conclusions

Novel fully biodegradable PBSMS/CNC composites were successfully prepared in this research at low loadings of CNC via a simple solution and casting process. CNC finely dispersed in PBSMS matrix at low loadings. CNC, as an efficient nucleating agent, obviously enhanced the melt crystallization of PBSMS. During the nonisothermal crystallization process, the *T*_cc_ of each sample significantly decreased with increasing cooling rate, while *T*_cc_ notably increased with increasing CNC loading at the same cooling rate. During the isothermal crystallization process, the *t*_0.5_ of PBSMS remarkably decreased with increasing CNC loading at the same *T*_c_. The nucleation activity and nucleation efficiency values of CNC were 0.83 and 23.3% in PBSMS/CNC0.5 and 0.70 and 43.5% in PBSMS/CNC1, respectively, revealing the good nucleation effect of CNC. In addition, the crystallization mechanism and crystal structure of PBSMS did not modify despite the presence of CNC. As a reinforcing nanofiller, CNC also obviously improved the mechanical property of PBSMS, as evidenced by the increased *E*_t_ and *σ*_y_. The improved crystallization and mechanical properties of PBSMS by CNC should be helpful for the practical application as a novel biodegradable polymer.

## Figures and Tables

**Table 1:** Summary of the Avrami parameters for PBSMS and its composites.

Samples	*T*_c_ (°C)	*n*	*k* (min^−*n*^)	*t*_0.5_ (min)
PBSMS20	76	2.2	2.54 × 10^−4^	36.6
	72	2.5	9.65 × 10^−4^	13.4
	68	2.2	6.49 × 10^−3^	8.5
	64	2.2	2.63 × 10^−2^	4.2
PBSMS20/CNC0.5	76	2.3	4.08 × 10^−4^	25.2
	72	2.4	3.02 × 10^−3^	9.0
	68	2.5	3.66 × 10^−2^	3.3
	64	2.5	2.32 × 10^−1^	1.5
PBSMS20/CNC1	76	2.7	7.21 × 10^−4^	17.9
	72	2.7	6.81 × 10^−3^	5.6
	68	2.6	1.40 × 10^−1^	1.8
	64	2.7	1.07	1.2

**Table 2:** Summary of mechanical properties of PBSMS and its composites.

Samples	*E* (MPa)	*σ_y_* (MPa)	*ε_y_* (%)	*σ_b_* (MPa)	*ε_b_* (%)
PBSMS	242.9 ± 12.4	10.6 ± 1.3	12.1 ± 0.7	12.7 ± 1.5	333.3 ± 54.6
PBSMS/CNC0.5	320.3 ± 14.6	16.5 ± 0.9	12.1 ± 1.1	13.8 ± 0.8	231.9 ± 15.6
PBSMS/CNC1	321.3 ± 5.14	19.1 ± 0.5	11.6 ± 1.4	13.9 ± 1.2	198.6 ± 14.7

## Data Availability

Data are contained within the article.

## Supplementary Materials

The following supporting information can be downloaded at: https://www.mdpi.com/article/10.3390/polym16121735/s1, Figure S1: DSC cooling curves of (a) PBSMS and PBSMS/CNC1 at different cooling rates; Figure S2: The heat flow evolution with crystallization time for PBSMS and its composites at 76 °C; Figure S3: Plots of relative crystallinity versus crystallization time for (a) PBSMS and (b) PBSMS/CNC1; Figure S4: Avrami plots for (a) PBSMS and (b) PBSMS/CNC1; Figure S5: DSC heating curves of the samples for the mechanical property test at 10 °C/min.
