# Supplementary material for: Crystallization Behavior and Mechanical Property of Biodegradable Poly(butylene succinate-co-2-methyl succinate)/Cellulose Nanocrystals Composites
Source: Polymers (Basel). 2024 Jun 19;16(12):1735. doi: 10.3390/polym16121735 (PMC11207285; doi:10.3390/polym16121735)
Supplement: Supplementary file 1 [file polymers-16-01735-s001.zip › polymers-3031891-supplementary.pdf]

Supplementary

The Crystallization Behavior and Mechanical Properties of Biodegradable Poly(butylene succinate-*co*-2-methyl succinate)/Cellulose Nan°Crystals Composites

Wenxin Yao, Siyu Pan and Zhaobin Qiu *

State Key Laboratory of Chemical Resource Engineering, Beijing University of Chemical Technology, Beijing 100029, China; 2022200363@grad.buct.edu.cn (W.Y.); 2022400087@grad.buct.edu.cn (S.P.)
* Correspondence: qiuzb@mail.buct.edu.cn

Preparation of PBSMS/CNC Composites

Two PBSMS/CNC composites were prepared through a solution and casting pr°Cess, which contained 0.5 and 1 wt% of CNC and were named as PBSMS/CNC0.5 and PBSMS/CNC1, respectively. Taking PBSMS/CNC1 as an example, the preparation pr°Cedure was described as follows. First, 2.97 g of PBSMS was dissolved into 35 mL of DMF after stirring at 35 °C for 2 h to form a solution, and 30 mg of CNC was dispersed into 15 mL of DMF with the help of the sonication at 350 W for 2 h (KQ-700DE ultrasonic generator) to form a suspension. Second, the PBSMS solution and the CNC suspension were further mixed and stirred at 35 °C for 4 h. Third, the solvent was evaporated at 35 °C in a fume hood to form a film. Finally, the obtained film was further dried in a vacuum oven at 50 °C for 7 days to completely remove the residual solvent. For comparison, neat PBSMS also underwent the same pr°Cess.

Nonisothermal Crystallization Behavior Study

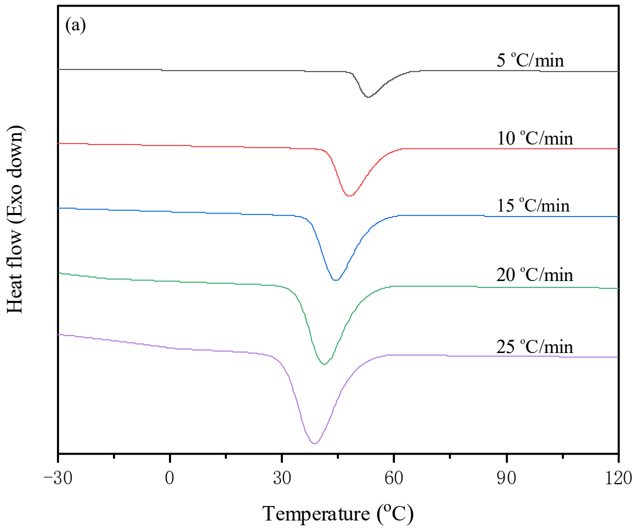

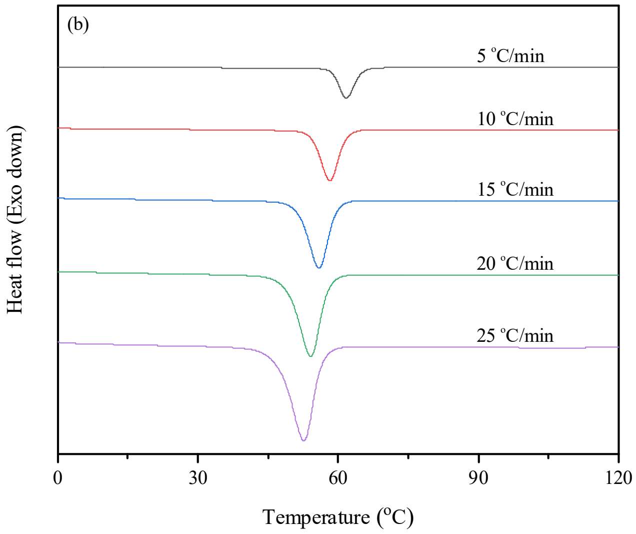

Figure S1. DSC cooling curves of (a) PBSMS and PBSMS/CNC1 at different cooling rates.

Isothermal Crystallization Kinetics Study

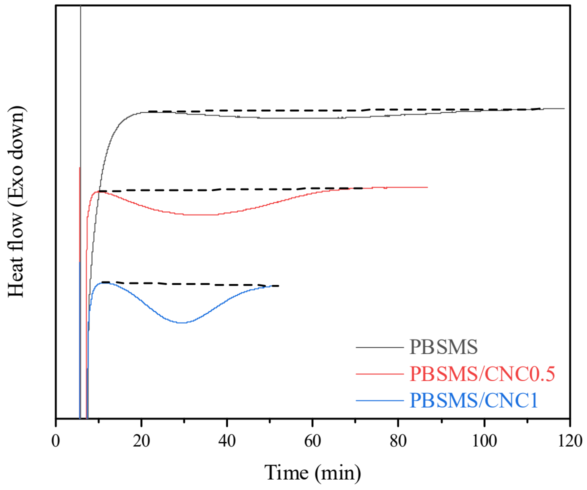

Figure S2. The heat flow evolution with crystallization time for PBSMS and its composites at 76 °C.

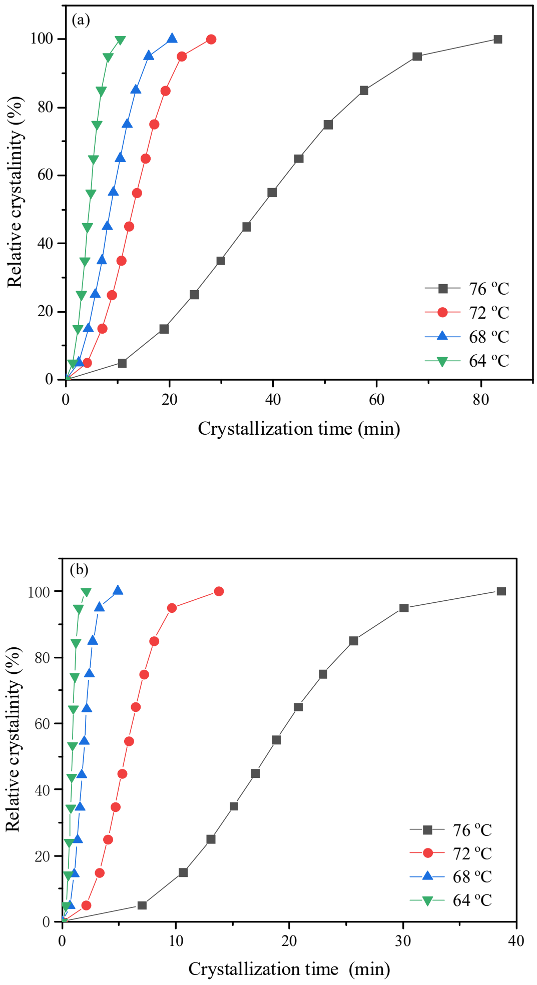

Figure S3. Plots of relative crystallinity versus crystallization time for (a) PBSMS and (b) PBSMS/CNC1.

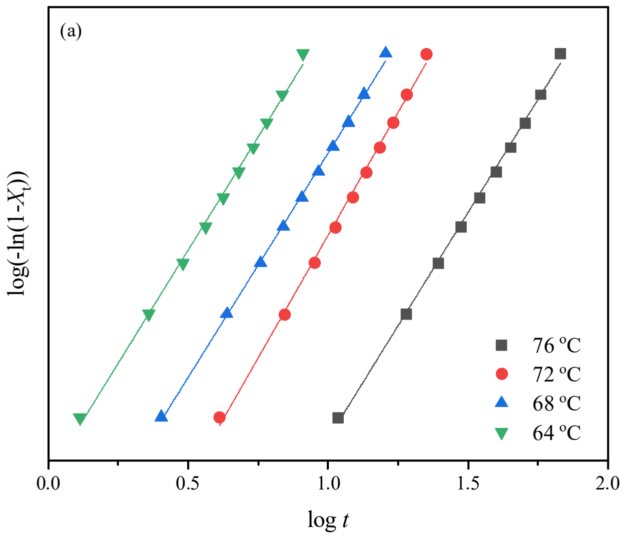

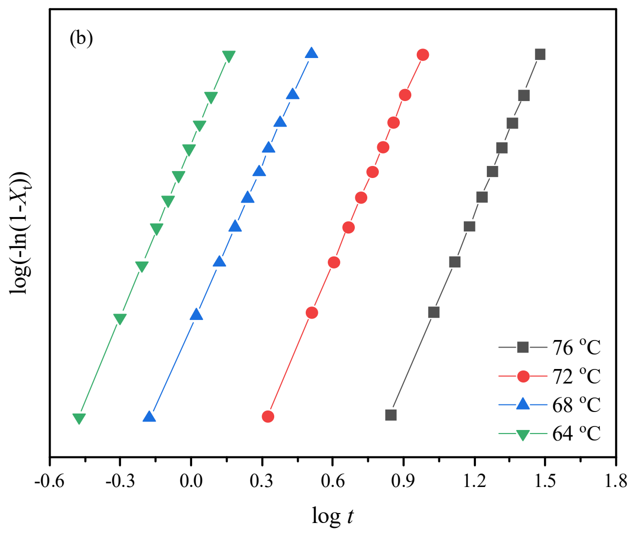

Figure S4. Avrami plots for (a) PBSMS and (b) PBSMS/CNC1.

Mechanical Property Study

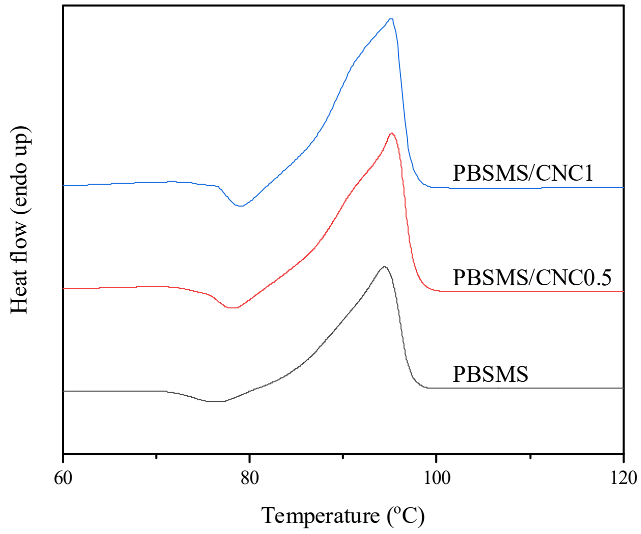

Figure S5. DSC heating curves of the samples for the mechanical property test at 10 °C/min.
